# Growth rate after limb deformity correction by the Ilizarov method with or without knee joint distraction: Lengthening in 30 children followed for at least 2 years

**DOI:** 10.3109/17453670903025345

**Published:** 2009-06-01

**Authors:** Hubert J Oostenbroek, Ronald Brand, Peter M van Roermund

**Affiliations:** ^1^Department of Orthopedics, University Medical CenterUtrechtthe Netherlands; ^2^Department of Orthopedics, Leiden University Medical CenterLeidenthe Netherlands; ^3^Department of Statistics, Leiden University Medical CenterLeidenthe Netherlands

## Abstract

**Background and purpose** Growth inhibition and stimulation have both been reported after juvenile limb lengthening. Distraction of a joint usually suspends and unloads the growth plate and may stimulate growth. We investigated the influence of knee joint distraction on the speed of growth after limb lengthening.

**Methods** In a retrospective study, growth patterns were analyzed in 30 children mean 61 (24–109) months after limb lengthening with the Ilizarov method, each child having more than 2 years of remaining growth. In 14 patients with knee joint instability, the knee was bridged over during lengthening for joint stabilization. Whether or not joint bridging and distraction would affect patterns of growth of the lengthened limb by unloading the growth plate was evaluated with a repeated measurements analysis of variance.

**Results** After lengthening procedures, the proportionate leg-length discrepancy was found to decrease in 16 children, suggesting increased growth rate in the lengthened limbs. A statistically significantly faster growth rate was seen in 8 of 14 patients with knee distraction as compared to patients with single bone frame configurations.

**Interpretation** Further research is required to investigate whether growth stimulation is due to the surgical technique and whether joint distraction should be recommended during limb lengthening in growing children.

## Introduction

The Ilizarov leg lengthening procedure is a well-established option in the treatment of limb-length discrepancy. Soft tissue tension resulting from the resistance of muscles, developed during distraction, may cause (sub)luxation and/or contracture in abnormal joints (Faber et al. 1991, Aldegheri 1999, Birch and Samchukov 2004). Moreover, due to this soft tissue tension, pressure forces on the adjacent physeal and articular cartilage may jeopardize the structure and function of these cartilaginous tissues, affecting growth and inducing degeneration of the joint cartilage (Wilson-MacDonald et al. 1990, Nakamura et al. 1995, Stanitski et al. 1996, Cai et al. 2006). To prevent such complications, a joint can be bridged and distracted during the lengthening procedure. Repeated joint distraction may prevent associated complications or even cause increased growth (Rajewski and Marciniak 1997).

We evaluated the possible long-term effect of lengthening and joint distraction on the growth pattern of the lengthened limb, as this may influence further decisions about treatment.

## Patients

30 children (16 girls) underwent lengthening procedures with the Ilizarov method (Table 1). 33 bone segments, 12 femoral and 21 tibial, were corrected. In 3 patients the femur and tibia were corrected simultaneously (patients 4, 11, and 25). The mean age at the start of the treatment was 10 (6–15) years.

**Table 1.** Characteristics of 30 patients prior to limb deformity correction, and description of their deformities

Patient Sex		Age (years)	Location **^a^**	Diagnosis	Concomitant deformities and factors **^b^**	LLD **^c^** (cm)	rLLD**^d^** (%)	Angulation and rotation (degrees)	Dahl type
1	F	9	T	growth arrest after osteo myelitis, proximal tibia	3,5,10,17,21	7.0	20	30 varus	4
2	M	12	T	congenital short tibia	3,9,24,24	5.6	16		4
3	F	9	T	femur-fibula-ulna syndrome	2,3,7,14,15,17	7.5	26	54 varus	5
4	F	6	T	femur-fibula-ulna syndrome	2,3,7,8,14,15,17,18,21	8.9	20	35 valgus, 45 int. rotation	5
5	F	9	T	congenital short tibia	6,8	6.1	23		3
6	F	9	F	congenital short femur	14	5.1	16		2
7	M	14	F+T	polyostotic fibrous dysplasia	2,3,12,18,21,22,22,26,26	17.9	20	45 int. rotation tibia	5
8	F	9	F	growth arrest after arthritis of the hip	1,3	4.3	13		2
9	F	11	T	child abuse, ischiac nerve lesion	11	4.5	20		2
10	M	14	T	congenital short tibia	3,7,8,9,15	4.9	15		3
11	M	8	F+T	congenital short femur + tibia	14	7.3	12		3
12	M	15	F	congenital short femur	17	4.5	9	20 valgus	1
13	F	13	F	growth arrest after fracture distal femur	2,4,10,17,18,21,21	4.4	11	10 valgus, 15 int. rotation	5
14	M	11	F	growth arrest after arthritis of the hip	1,2,17,21,21,22	4.2	11	20 procurvatum	4
15	F	7	T	congenital short tibia	5,7,8,15,22,23,24	7.8	26		5
16	M	6	T	congenital short tibia	2,5,7,8,14,15,22,23	8.4	36		5
17	M	11	T	Ollier's disease	2,17,18	4.6	16	35 ext. rotation, 18 retrocurvatum	4
18	M	8	F	congenital short femur	19,22,25	5.4	18		3
19	F	9	F	growth arrest after arthritis of the hip	17	2.9	8	20 valgus	2
20	F	10	T	congenital short tibia	3,17	5.4	18	14 valgus	3
21	M	14	T	growth arrest after arthritis of the knee	17	3.9	7	20 valgus	2
22	M	7	T	congenital short tibia	3,7,14	8.9	33		4
23	F	6	T	congenital short tibia	3,7,8,14,17	10.2	42	25 procurvatum	5
24	F	8	T	congenital short tibia	7,13,16,17,21,21,22,24	1.7	6	30 procurvation	4
25	M	11	F+T	congenital short femur +tibia	14	7.7	10		3
26	F	11	F	growth arrest after arthritis of the hip	2,3,14,20	14.8	38		5
27	M	7	T	femur-fibula-ulna syndrome	2,3,7,8,14,15,22,23	6.2	29		5
28	F	10	T	Ollier's disease	3,7,15,17,21	2.6	9	35 valgus	2
29	F	13	T	congenital short tibia		3.4	8		1
30	M	9	F	congenital short femur	7,15,17	1.9	7	30 valgus	

**^a^** Location: F – femur; T – tibia.

**^b^** Concomitant deformities and factors according to Dahl et al. (1994): 1 contracture hip, 2 contracture knee, 3 equinus, 4 ankylosis knee, 5 ankylosis ankle, 6 tarsal coalition, 7 fibular hypo-/aplasia, 8 absence of foot rays, 9 clubfoot, 10 dislocated patella, 11 ischiatic nerve lesion, 12 femoral pseudarthrosis, 13 tibial pseudarthrosis, 14 ACL aplasia, 15 ball and socket ankle, 16 active infection, 17 angulation deformity, 18 torsion deformity, 19 hip dysplasia, 20 lateral femoral condyle dysplasia, 21 previous lengthening procedure, 22 previous correction osteotomy, 23 previous resection fibular fibrous band, 24 previous correction equinus deformity, 25 other previous soft tissue corrections, 26 simultaneous correction in two bone segments.

**^c^** LLD (cm): leg-length discrepancy (LLD) in centimeters.

**^d^** rLLD (%): relative leg-length discrepancy; see text for the definition.

**^e^** Dahl (type): see text for definition of the Dahl types regarding the severity of the deformity.

### Deformity and classification

The mean preoperative leg length discrepancy (LLD) was 6.3 (1.9–18) cm, and the mean percentage LLD was 18 (6–42).

The severity of the deformities was classified into 5 types according to Dahl et al. (1994). Type 1 indicates less than 15% LLD; type 2: 16–25%; type 3: 26–35%; type 4: 36–50%; and type 5: more than 50% LLD. The type of severity increases one level when 2 greater risk factors (e.g. congenital origin of the deformity, previous lengthening, multisite correction) are present, and when 3 lesser risk factors (e.g. pre-existing joint contracture, neurological deficit, location of the deformity in the femur or foot) are present. The deformity in our study population was classified as type 1 in 2 children, as type 2 in 7, as type 3 in 6, as type 4 in 6, and as type 5 in 9 children.

## Methods

Preoperatively, the length discrepancy was calculated from a single length measurement, which is sufficient for an accurate prediction of the future leg length discrepancy (Aguilar et al. 2005). The measurement was made on standing AP radiographs, which are reliable for length measurements (Sabharwal et al. 2007).

In all procedures, bone lengthening was performed by callus distraction with an Ilizarov ring fixator after a corticotomy. At the end of the operation, to prevent (sub)luxation, contracture, or potentially harmful pressure on articular and physeal cartilage (due to high tensile forces found in the soft tissues following lengthening (Cai et al. 2006)), knee joints were bridged and the knees were distracted in 14 children for about 1–2 mm after application of the frame, under direct fluoroscopic control. After corticotomy, distraction was delayed for 5–7 days. Distraction was 0.25 mm, 3–4 times a day.

In the outpatient clinic, the children were seen at 2- to 3-week intervals during lengthening and every 4–6 weeks during the consolidation phase. Joint distension of approximately 2 mm was controlled on the radiographs at every visit. If there was any reduced distension, the joint was distracted to such an extent that the primary radiographic joint distension was regained. This procedure was repeated as required during the whole period of frame application. After radiographic evidence of consolidation of the distraction callus, the frame was removed. Cast immobilization was applied for 2–4 weeks and a brace was given for another 6–8 weeks. Weight bearing was encouraged during the treatment period and physiotherapy was given. If needed, psychological support was provided to the child and the family.

### Growth pattern

At least 2 orthoradiographs for leg-length measurements were performed after removal of the Ilizarov frame, to evaluate further growth in length in both lower limbs. These measurements were performed after mean 61 (24–109) months. All patients had more than 24 months of remaining growth after Ilizarov treatment. The decision to select this time was because bony interventions or fractures of the limb may lead to locally, although temporarily (less than 2 years) increased growth (Stephens et al. 1989, De Sanctis et al. 1996). Since we wanted to know whether knee joint distraction had any additional effect on the growth pattern, we had to look beyond 2 years after the start of the treatment.

To calculate the growth of the treated limbs, gain in length from the distraction—calculated between osteotomy ends—was deducted from the leg-length measurements after the Ilizarov procedure. Accordingly, any change in the proportionate (%) limb length discrepancy would identify a change in the growth rate.

### Data analysis

Aguilar et al. (2005) reported that growth patterns can be predicted very accurately with a single limb-length measurement by a multiplier method. From these data, we interpreted that limb-length discrepancy expressed as the ratio of the length of the long (usually normal) limb and of the shortened limb is a constant measure. In a graph in which the x-axis represents the length of the long (normal) leg and the y-axis represents the length of the shortened leg, the constant ratio (proportionate LLD) is expressed by a straight line with a slope identical to the ratio. After intervention, the slope of this line may change—in the sense that increased growth of the lengthened leg results in a steeper line (negative intercept, of the line with the y-axis) and reduced growth results in a shallower line (positive intercept, of the line with the y-axis) (Figure).

**Figure:**
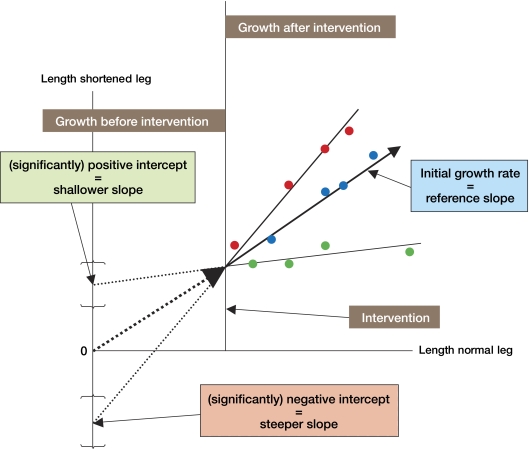
Graphical representation of the statistical principle of a positive and negative intercept as the result of an intervention in a shortened leg, compared with the normal leg. The length of the normal leg is represented on the x-axis. The length of the shortened leg is represented on the y-axis. The reference line represents the relationship between the lengths of the normal and shortened legs. The relationship between the lengths of normal and shortened legs may change, e.g. by surgical intervention. For better graphical representation, the gain in length from the lengthening procedure is deducted from the length of the shortened leg after the intervention. When the line of proportionate growth shows a changed slope after the intervention, as compared to the reference line, and when this new line has a negative intercept with the y-axis, it represents increased growth of the shortened leg compared to the normal leg. When this new line has a positive intercept with the y-axis, it represents decreased growth of the shortened leg compared to the normal leg.

Since we only have one measurement before the intervention, the slope of the line before the intervention is not known. However, due to the reasonable assumption that the growth ratio is constant, considering the very accurate multiplier findings of Aguilar et al. (2005), this line should go through the origin. Hence, as a proxy to the test whether the slopes before and after intervention are different, we can simply test whether the regression line after intervention has an intercept above or below the origin (corresponding to a reduced or increased growth speed, respectively).

Data were analyzed by a repeated-measurements analysis of variance using a mixed model in SPSS version 12.0.1. Repeated measurements were obtained over the follow-up time. The subject is a random factor within which multiple observations (follow-up moments) are nested. The normal limb is entered as a covariate (the x-axis) and the other limb's length is used as outcome variable. Each child contributes a varying number of observations over the follow-up time. All factors except the subject were taken as fixed effects. Thus, the estimated slope and intercept were calculated as a common, fixed effect. We did not assume any specific model for the outcome itself, but the correlation of repeated measurements was assumed to be of autoregressive type (order 1). A p-value of less than 0.05 was considered statistically significant. The primary parameter of interest was the estimated intercept itself and not the slope of the regression line (see Figure and the explanation given above).

We analyzed different variables that may influence growth. Type of frame (mono-osteal: femur or tibia compared with poly-osteal with bridged and distracted knee), location of osteotomy (femoral, tibial, or both), and type of deformity (congenital, acquired). These variables were entered into the model as categorical covariates. In the case of 3 or more categories, an overall test was first performed; only if that effect was significant, multiple comparisons were performed to compare the various subgroups. All effects and the associated 95% confidence intervals were estimated from these models.

## Results

The mean lengthening was 4.8 (1–8) cm, and the mean percentage lengthening was found to be 14 (3–28). The mean lengthening index was 1.5 (0.8–3.3) months per cm of lengthening (Table 2).

**Table 2.** The results of limb deformity correction in 30 patients

Pat	Frame **^a^**	Ost. site **^b^**	Consol. **^c^** (weeks)	Paley complications **^d^**			Angulation	Gain (cm)	Gain (%)	LI **^e^** (mo/cm)	LLD **^f^** (cm)	rLLD **^g^** (%)	F-U **^h^** (mo)	ΔLLD po **^i^** (cm)	Growth rate **^j^**
				problem	obstacle	real									
1	3	2	42	8				6	17	1.7	13	37	73	3	–
2	2	2	30	4,8				6	17	0.8	0.6	2	60	0.1	+
3	3	2	38	8	2	3,9,9	50° varus	6.5	23	1.4	4.6	18	40	4.5	–
4	3	3	22	8	1,6			5	11	1.0	7.8	30	103	0	+
5	2	2	23		8			6	23	0.8	0	0	97	2	+
6	1	1	26	1,8		10		5.5	17	1.0	1.7	4	45	3.3	–
7	3	2	19	4,8,8		9		6	7	1.1	14	15	41	6.4	–
8	1	1	28	7,8		3	14° valgus	5	15	1.2	0.7	2	77	-0.3	+
9	2	2	26	8				4	18	1.5	1.7	5	47	0.5	o
10	2	2	28	4				5.5	17	1.2	3.8	9	33	-0.7	+
11	3	3	31		9	9		8	13	0.9	-0.7	-1	109	0.6	+
12	1	1	23	8				4	8	1.3	0.5	1	28	0	+
13	3	1	32	7,8				4	10	1.9	1	2	24	0	+
14	3	1	27	8				5.5	14	1.1	0.5	1	72	1.5	–
15	2	2	29	8		6,9		4.5	15	1.6	4.7	14	83	-0.7	+
16	3	2	25	8				6	26	1.0	2.5	10	32	2.3	o
17	2	2	8	7,8		3,4,6	16° retrocurvatum	1	3	2.0	4.4	15	64	5.9	–
18	1	1	21	8				5	17	1.0	-0.5	-1	99	3.5	–
19	1	1	22	7	8	3	20° procurvatum	2	6	2.0	2	5	60	3.1	–
20	2	2	32	8				5	17	1.5	0.5	2	55	2	o
21	2	2	20	8		6		2	4	2.5	2.7	7	24	0.1	+
22	3	2	27	8				6	22	1.0	4	14	63	0.4	+
23	3	2	25					6	25	1.0	4.2	12	96	1.7	+
24	3	3	11	8				1	4	2.5	0.9	3	65	4.3	–
25	3	3	30	8	8	10		8	10	1.0	0	0	66	0.6	+
26	3	1	30	8				6	15	1.1	8.4	21	48	2.1	+
27	3	2	36	8				6	28	1.0	0	0	62	0.5	+
28	2	2	31			3,6	10° valgus	2	7	3.0	0	0	102	0	o
29	2	2	33			9		3	7	2.6	1	2	49	1	+
30	1	1	28					2	7	3.3	0	0	28	0	o

**^a^** Frame: configuration of Ilizarov frame: 1 femur, 2 tibia, 3 femur and tibia.

**^b^** Ost. site: osteotomy site: 1 femur, 2 tibia, 3 femur and tibia.

**^c^** Consol.: consolidation time in weeks, duration of correction and consolidation, total period in frame.

**^d^** Paley complications: classification of complications (Paley 1990): problems (difficulties resolved without operation), obstacles (difficulties resolved with operative intervention), minor and major complications (all intraoperative injuries; difficulties not resolved before the end of the treatment, minor complication if resolved with nonoperative treatment, major complication if operative treatment is required): 1 muscle contracture, 2 joint luxation, 3 axial deviation (minor < 5º, major > 5º), 4 neurological injury (peroneal nerve), 5 vascular injury, 6 premature consolidation, 7 delayed consolidation, 8 pin-site problems, infections, 9 refracture, 10 joint stiffness.

**^e^** LI: lengthening index; period in frame (months) divided by length gained (cm).

**^f^** LLD: residual leg length discrepancy in cm.

**^g^** rLLD: residual relative leg length discrepancy, see text for the definition of relative LLD.

**^h^** F-U: follow-up in months after initiation of treatment.

**^i^** ΔLLD po: difference of LLD from first and last leg length measurement in cm.

**^j^** Growth rate: + stimulated; o neutral; – decreased growth rate.

### Growth patterns

In 16 children, the proportionate shortening of the shortened leg decreased. Five children had stabilized proportionate shortening and 9 children had increased proportionate shortening. Frame configuration is the most important factor for growth rate. Frames with knee bridging were associated with an increased growth rate as compared to mono-osteal frames (Tables 3 and 4A). Even so, not all children with knee bridging experienced growth stimulation (only 8 of 14 children): 1 patient had unchanged growth and 5 of the 14 children with knee distraction showed growth inhibition. Cause of LLD and site of osteotomy had no statistically significant association with growth patterns of the leg, except for tibial growth, which was significantly different after tibial osteotomy (Tables 3 and 4B). All osteotomies caused growth inhibition, but tibial osteotomy caused almost none compared with femoral osteotomy or combined femoral and tibial osteotomy.

**Table 3.** Results of statistical testing using a repeated-measures ANOVA mixed model: significance of the possible treatment factors that may influence the pattern of growth of the limb. The results of significant factors are considered in Table 4

	P-value
Frame configuration, on the whole leg	0.003
Location of osteotomy, on the whole leg	1.0
Cause of deformity, on the whole leg	0.8
Location of osteotomy, on the femur	0.09
Location of osteotomy, on the tibia	0.04

**Table:** Table 4A. Results of statistical testing using a repeated-measures ANOVA mixed model. The frame configuration has a significant effect on growth pattern. The negative value of a knee-bridging intercept indicates a decreasing proportionate leg length discrepancy, i.e. growth stimulation

Frame configuration: p = 0.003		95% CI
Intercept, femoral frame	3.1	(-2.0–8.2)
Intercept, tibial frame	3.3	(-1.5–8.1)
Intercept, knee-bridging frame **^a^**	-1.7	(-6.1–2.6)

**^a^**This category differs significantly from the other two categories

**Table:** Table 4B. Results of statistical testing using a repeated-measures ANOVA mixed model. The location of the osteotomy for the lengthening procedure has a significant effect on the growth pattern. The positive intercept indicates an increasing proportionate leg length discrepancy, i.e. inhibited growth for all types of osteotomy. The tibial osteotomy has significantly less inhibitory effect than other types of osteotomy

Location of osteotomy on the tibia: p = 0.04		95% CI
Intercept, femoral osteotomy	4.0	(0.5–7.6)
Intercept, tibial osteotomy **^a^**	0.7	(-9.9–11.0)
Intercept, osteotomy of femur and tibia	3.6	(-2.6–4.1)

**^a^**This category differs significantly from the other two categories.

## Discussion

The finding of increased growth after a limb lengthening procedure has been rarely reported. Increased growth has already been registered in 1 patient after a limb lengthening procedure (Sharma et al. 1996) and for 2 other lengthened legs, the feet were reported to grow stronger after the lengthening procedure (Saleh and Goonatillake 1995). Growth stimulation has been reported in a limited number of patients after femoral lengthening in 2 studies (Shapiro 1987, Sabharwal et al. 2000). Group effects have not been reported (Shapiro 1987, Hope et al. 1994, Sabharwal et al. 2000, McCarthy et al. 2003). It is known that after a femoral fracture, growth in the length of the traumatized leg may increase temporarily, but this effect always lasts less than 2 years after the trauma (De Sanctis et al. 1996, Stephens et al. 1989).

The definition of growth stimulation is important to explain our results. Usually, untreated shortened limbs show an increasing absolute shortening due to a general inhibition of the growth. At the same time, the proportionate (percentage) length discrepancy remains unchanged. Growth stimulation of the shortened leg is a change in the growth trend that results in a decreasing proportionate length discrepancy. When there is a mild proportionate stimulation of growth, the absolute shortening may increase during further growth simultaneously. Our finding of an increased growth rate for more than 2 years is not easy to explain. Our reason for unloading of the growth plate and joint cartilage was to protect these tissues from compressive forces by knee joint distraction during the lengthening procedure. This concept is supported by the results of an experiment in rabbits with Achilles tenotomy to unload the tibial growth plates during lengthening (Sabharwal et al. 2005). Gradual distraction of the knee joint may act as a mild form of chondrodiastasis, as suggested by De Bastiani (De Bastiani et al. 1986). Change in the growth program of the physis is induced by an unknown mechanism.

We visually controlled distraction with fluoroscopy during the operation, and later on with radiographs at intervals. We were unable to measure the compression or distraction forces acting upon the physes, so we could not verify whether the unloading of the physes was maintained continuously. One reason for not all children experiencing an enhanced growth rate may be that we were not able to control the joint distraction on a continuous basis. Uncertainty remains as to whether growth patterns are predictable or variable, especially after lengthening or other interventions. Aguilar et al. (2005) showed that growth patterns can be calculated accurately and predicted with a single preoperative length measurement, although they suggested that multiple measurements may lead to more accurate predictions. Paley et al. (2004) showed that growth patterns remain constant, and are independent of diagnosis, treatment, race, continent, historical period, chronological age, and skeletal age (Paley et al. 2004). These data were compared to the gold standard of the Anderson and Green data (Anderson et al. 1963), but also to many other databases of clinical and anthropological measurements. Consequently, it must be assumed that growth patterns remain predictable after lengthening, after epiphysiodesis, in congenital limb-length discrepancy, and in skeletal dysplasia, because there is little variation in the outcome. As has been shown by several authors, this does not apply to growth patterns after fractures; in this situation, growth is only temporarily stimulated and becomes normal in less than 2 years (Stephens et al. 1989, De Sanctis et al. 1996).

Unreliability of length measurements may influence the growth patterns seen. Even so, in centers with experienced personnel such as ours, the reliability of the measurements is usually within a few mm (Sabharwal et al. 2007). This corresponds to about 1% error in proportionate LLD, because it represents between 2 and 5 mm of bone length (depending on the bone length: 20 cm for very short tibias and 50 cm for normal femurs). So, the calculated average change of 4% in our children means between 8 and 20 mm. If we take into account that lengthening in many cases causes further growth retardation (Sharma et al. 1996, Viehweger et al. 1998, Sabharwal et al. 2000, McCarthy et al. 2003), we have observed a remarkable effect.

Further studies are required to confirm our findings of enhanced growth after the use of knee joint distraction during lengthening procedures, and to find out whether bridging and distracting the knee joint can be recommended in Ilizarov treatment to prevent complications and to stimulate physeal growth. Joint distraction as a single treatment in growing individuals may be considered in the future for the treatment of limb-length discrepancy, and should be investigated. Continuous monitoring of the forces acting upon the physes may be an important parameter to investigate, because we found indirect evidence that unloading of the physes causes stimulated growth. Decreasing proportionate length discrepancy as a biological phenomenon is intriguing and difficult to explain.
